# The American Sanitary Association

**Published:** 1895-03

**Authors:** 


### The American Sanitary Association.

The American Sanitary Association has been formed. W. Thornton Parker, M. D., Secretary, Groveland, Mass., has issued the following:

OBJECTS: To aid in the maintenauce of public health; to discourage the manufacture and sale of impure and injurious foods and medicines; to encourage the introduction of wholesome and honestly manufactured articles of foods, medicines, clothing, and sanitary appliances in general; to examine and certify as to the character of foods, medicines, and sanitary appliances; to distribute, for the benefit of the general public, information concerning sanitary matters.

Medical men, sanitarians, and others interested in public health, are cordially invited to become members. The enrollment fee is $2.00. Annual dues, $1.00.

